# Long Arm of Motorway—The Impact of Fenced Road on the Mortality of European Badgers

**DOI:** 10.1007/s00267-021-01570-y

**Published:** 2021-11-29

**Authors:** Krzysztof Nowakowski, Agnieszka Ważna, Przemysław Kurek, Jan Cichocki, Jacek Bojarski, Grzegorz Gabryś

**Affiliations:** 1 Department of Zoology, Institute of Biological Sciences, University of Zielona Góra, Prof. Z. Szafrana 1, 65-516 Zielona Góra, Poland; 2 Department of Plant Ecology and Environmental Protection, Adam Mickiewicz University, Uniwersytetu Poznańskiego 6, 61-614 Poznań, Poland; 3 Department of Mathematical Statistics and Econometrics, Institute of Mathematics, University of Zielona Góra, Prof. Z. Szafrana 4a, 65-516 Zielona Góra, Poland

**Keywords:** European badger, Road mortality, The road network development

## Abstract

We studied the impact of the new fenced and accident-safe motorway on the mortality of European badgers *Meles meles* on local roads in western Poland in 2010–2015. We monitored the badgers mortality on local roads of three categories: main roads, secondary roads and county roads. The study was conducted before and after the opening of the motorway in 2012. We hypothesized that the mortality of badgers is lower due to traffic concentration on motorway. Ninety two badgers were killed in collisions with vehicles on all monitored roads. Mean number of killed badgers was lowest in 2010 before the motorway opening and the highest in 2012. The mortality of badgers on regional roads was highest after the opening of the motorway due to the changes in traffic on the access roads. Within the road network, the mortality of badgers was 5.8 individuals/10 km of road per whole study period with the highest rate on main roads 8.5 individuals/10 km. The badgers mortality was highest on county roads but it was lower than expected in relation to the road network density. The highest vehicle collision risk for badgers of both sexes occurred in June. Distance to human settlements was the only environmental factor that was positively related to badger mortality on roads. We conclude that the new motorway did not reduce the mortality rate of badgers on the adjacent roads because the status of local roads has changed and now they mainly function as access roads to the motorway.

## Introduction

Animal mortality in collisions with vehicles on roads is an effect of an increase in road infrastructure of global importance (e.g., Hels and Buchwald 2001; Forman et al. 2003; Ruiz-Capillas et al. 2015; Hill et al. 2019; Schwartz et al. 2020; Grilo et al. 2021). Mortality rates on roads may be attributed to various factors related to the greater activity of animals in road corridors, such as crossing the roads from home ranges located in the neighborhood of roads or using a road as a feeding ground (Trombulak and Frissell 2000; Jaarsma et al. 2006; Sabino-Marques and Mira 2011; Hill et al. 2021).

An increase in mortality due to collisions with vehicles is one of the most important aspects of the effect that roads have on local populations of animals. This problem occurs all over the world and in all ecosystems, from highly urbanized areas to natural ecosystems, and involves many species. The traffic flow are the main causes of habitat fragmentation (Trombulak and Frissell 2000). The estimation of the influence that roads have on the ecosystem is very difficult at the planning stage. It also constitutes a great challenge when it comes to the impact of roads within the context of the whole road network. The scale of the problem may differ depending on many factors, such as the type of the road, road network density, distance to urban areas, vegetation type along the road, elevation above sea level, climate, season of the year, and human population density (Borkovcová et al. 2012; Hothorn et al. 2012; Baigas et al. 2017; Visintin et al. 2016). Animal mortality is also influenced by the following factors: traffic flow, traffic volume, the density of vehicles and the speed of the vehicles. The traffic volume and traversing speed have the greatest influence on the mortality of mammals in collisions (van Langevelde and Jaarsma 2004; Dennehy et al. 2021). Nevertheless, the increase in traffic volumes on the existing roads still has smaller impact on mortality than road construction (Rhodes et al. 2014).

Mortality on roads is an issue for populations of many mammal species. Carnivores are evidently among those animals at highest risk of collision with vehicles. Stone marten *Martes martes*, European otter *Lutra lutra* and red fox *Vulpes vulpes* are the most frequent casualties from this group (Červinka et al. 2015), but in those areas where large carnivores occur, wolf *Canis lupus*, Eurasian lynx *Lynx lynx* and brown bear *Ursus arctos* they have also been reported killed on roads (e.g., Kusak et al. 2000; Lovari et al. 2007; Boulanger and Stenhouse 2014).

Collisions with vehicles contribute significantly to the mortality of European badgers *Meles meles*. In European countries, hundreds of badgers are killed on roads. Specifically, in the UK (Davies et al. 1987; Clarke et al. 1998), Netherlands (Jaarsma et al. 2007; van Langevelde et al. 2009; Dekker and Bekker 2010), Denmark (Aaris-Sørensen 1995), Ireland (Sleeman et al. 2009; Haigh 2012; Sleeman et al. 2012), Czechia (Červinka et al. 2015), Lithuania (Balčiauskas et al. 2020) and Italy (Valerio et al. 2021). The mortality of badgers on roads has also been reported in Poland (Kowalczyk et al. 2003; Orłowski and Nowak 2006; Jakubas et al. 2018; Nieszała and Klich 2021).

The development of the road network in Poland has intensified since the end of the 1990s, mainly due to subsidies from the European Cohesion Fund (Ślusarczyk and Broniszewska 2013). In western Poland, the expansion of expressways and motorways is advanced and most of the investments have already been completed or their completion is imminent. Regardless of the development of road connections in Poland, a serious increase in road traffic is observed on main national roads (GPR 2015). Local road traffic is not monitored but it has recently increased visibly due to the increase in the number of cars per household. In 2006–2016, the number of passenger cars in Poland increased by 55%, which means that the number of cars per 1000 passengers (564 cars) exceeds that for France and the United Kingdom (unece.org).

Both the development of the road network and the increase in road traffic imply that it is necessary to analyze the impact of these factors on the natural environment, especially on animal populations. In the areas particularly exposed to collision, various safety measures are applied to reduce animal mortality, e.g., road fencing, wildlife crossings or speed limits (e.g., Jaeger et al. 2005; Ascensão and Mira 2007; McCollister and van Manen 2010; Huijser et al. 2016; Plante et al. 2019; Brunen et al. 2020; Spanowicz et al. 2020). Mitigation measures have different effectiveness, the highest being road fencing (Rytwinski et al. 2016). Economical factor has a significant meaning in this situation and road fencing is the most effective in preventing from collisions in case of main roads characterized by the highest traffic intensity (Kučas and Balčiauskas 2021). In Poland, all newly built motorways and expressways are fenced and have different types of wildlife passages (e.g., Mysłajek et al. 2020; Ważna et al. 2020). The mortality of large- and medium-sized mammals on these roads is very low. However, the impact of the newly built motorway on animal mortality on the neighboring roads is unknown.

The aim of this study was to evaluate the changes in the mortality of badgers in relation to various environmental factors, seasons of the year and different categories of roads in western Poland before and after the opening of A2 motorway. We collected data over 6 years, starting two years before and ending four years after the opening of the motorway. In comparison to other categories of roads, motorways concentrate a huge part of the traffic. To minimize the risk of collisions with wild animals, all motorways in Poland are fenced to hinder the unpredictable access for animals. Thus, despite higher traffic and high speed rate of vehicles on motorways, these roads seem to be much safer for wild animals than other types of roads that are more easily accessible (no fences applied). We hypothesized that (1) the mortality of badgers on regional roads after the opening of a motorway is lower due to high-velocity traffic concentration on fenced and accident-safe motorway, (2) mortality of badgers is higher on roads within forests than in adjacent farmland areas, (3) the mortality of badgers depends on sex and it is higher during mating time when the mobility of badgers increases.

## Materials and methods

### Study Area

This research was conducted in the lowland landscape of western Poland. The study area covered 389.52 km^2^ (52°17′–52°32′ N, 15°30′–16°01′ E). Forests cover 52% of the area and consist of 213 complexes of 1–2000 ha. Coni-ferous forests constitute 73% of the general forest area, deciduous forests - 22.8%, and wet ash-alder forests cover 4.2%. Non-forest areas outside urbanized areas consist of agricultural lands and meadows. Four rivers - Obra, Leniwa Obra, Czarna Woda, and Paklica - flow through the study area. The total area of lakes is 1350 ha.

The public roads network in Poland comprises four road categories: national roads (motorways, express roads, nonfenced main roads), secondary roads (regional roads), county roads and municipal roads. The national roads are managed by General Directorate for National Roads and Motorways. The roads of lower categories are managed by local governments (Ministerstwo Infrastruktury 2021). The road network under this study embraced roads of various categories, depending on their importance, according to the system accepted in Poland. We considered three different categories present within our study site: A - main road (not fenced national road) of international and national importance (27 km) creating a route for transit traffic through Poland from west to east, used as a free alternative to the toll motorway. It is a two-lane road with reinforced shoulders, not fenced. In 2014, the average traffic volume on this route amounted to 9267 vehicles/day (ARD 2015); B - secondary road (regional road) of national and regional significance (16 km), not fenced - a route running from the north to the south of the country. It is a two-lane road without reinforced shoulders. In 2015, the average traffic volume on this route was 1351 vehicles/day (GPR 2015); C - county roads (municipal roads) of regional and local significance (115 km), not fenced. These roads form a network of roads connecting cities within the region. These are two-line roads without reinforced shoulders. No traffic measurements are carried out on roads of this category. The roads do not differ in the speed limit. The total length of the roads in the study was 158 km. Badger mortality data were assigned to each category of the road. Badgers did not have access to motorway A2 that crosses the study area due to the protection fencing, therefore badger mortality was not monitored on that motorway.

### Data Collection

We studied badger mortality within the entire road network associated with A2 motorway from 2010 to 2015. We used personal observation of road shoulders during regular inspections by car. The category A road was monitored daily due to the high traffic intensity that exposed animal carcasses to destruction and due to the irregular checks by road services that cleared dead animals from the roadway. The B and C category roads were inspected once a week year round and each time we obtained information about collisions from our informers. The B and C roads are not patrolled by road services. We also collected information from witnesses of collisions (hunters, forest workers). Information about the study was provided in local media and on a popular forest internet blog. All data obtained from indirect sources (blog, witnesses) were checked and confirmed during field inspections. During the inspections we found 44 badgers carcasses and 48 victims of the collisions were reported from other sources.

We examined badger carcasses for sex, age and other characteristics (e.g., visible pregnancy, signs of lactation). We estimated the age of badgers based on tooth characteristics. Animals were divided into three groups: (a) ≤ one-year old, (b) one to two years old, and (c) >two years old (Stubbe 1965; 1970). We did not estimate biological parameters in seriously damaged individuals. After examination, we removed dead badgers from the road. If no dead animal was found, despite precise information from a witness, the collision was confirmed based on biological evidence such as blood or hair. In such cases, only the occurrence of the collision was noted and environmental parameters were recorded.

The risk of collision may be linked to the search for water and food or to penetration of the burrow area. Thus, for all collision sites, we measured the nearest distance to: (a) inhabited main badger sett, (b) water source (i.e., ditches, canals, rivers, fish ponds, lakes), (c) human settlement and (d) forest. Also, the distribution of collision sites depending on road category (A – main, B – secondary, C – county) was considered in the analysis. The distance from the nearest inhabited badger sett, water source, human settlements and forest were estimated on the basis of 98 random points generated by the computer with the use of digital forest maps. The distances were measured on the basis of GPS data of the collision locations and the location of the setts and the nearest landscape points. During the study period, main setts were monitored to estimate the nearest distance to the inhabited sett. We assessed the state of sett utilization based on the traces left by animals around the entries: tracks, digging and presence of bedding material.

### Statistical Analysis

**Fig. 1:**
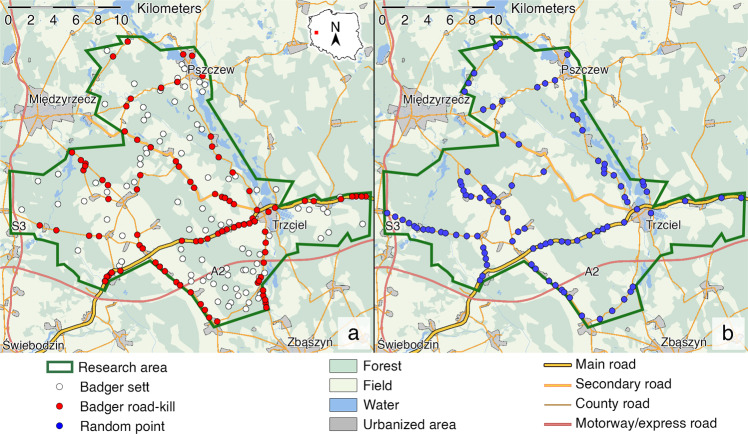
Location of badgers killed on roads in western Poland in 2010–2015 (**a**) and random points used for environmental analysis of badgers mortality (**b**)

A total of 190 data points were analyzed, 92 killed badgers and 98 random points on roads designated using Excel RAND Function (Fig. 1). According to low activity of badgers and thus low collision rate in winter time only data concerning collisions from March-October (*N* = 89) were considered for ANOVA analysis. Factorial ANOVA was used to estimate the significance in differences between the number of killed badgers including year and month as factors. In the case when interaction year × month was insignificant it was excluded from the analysis and only factors were tested. To distinguish the effect of the opening of motorway A2 on the rate of killed badgers, data concerning the number of victims in consecutive years of the study were divided into two groups – before (years 2010, 2011) and after (years 2012–2015) the opening of motorway A2. There were no significant differences in mean values between years within the longer period of the study, which was after the opening of the motorway (2012–2015), so other factors than a motorway itself that might have impacted the increase in collisions, such as the increased traffic, could be excluded. Thus, it allowed the comparison of two periods of time that differed in duration.

Prior to all analyses, the skew data were transformed with logarithmic or exponential functions to obtain a normal or at least symmetric distribution. For environmental linear data, generalized linear models (GLMs) for binomial distribution of dependent variable (1 – dead badger, 0 – random point) were used to explain the variation of badger mortality. We predicted that the distance from the nearest inhabited sett, water source, forests and human settlements have impact on probability of collisions with badgers (explanatory variables). There was no colinearity between explanatory variables (max GVIF < 3). Transformed data were scaled before analysis. Residuals versus expected values and overdispersion were checked with DHARMa package (Hartig 2020). The model selection was provided by the MuMIn package (Bartoń 2020). Selected models were ranked on AICc values and simple models with ΔAICc values <2 were considered for further computations (Burnham and Anderson 2002). For categorical data (road type) the chi-square test of independence (χ^2^) was used to determine the differences between groups of spots with a killed badger in proportion to variable availability expressed with random points. All analyses were performed with RStudio (RStudio Team 2021).

## Results

During the 6-year study (2010–2015), we found 92 badgers killed in collisions with vehicles. For all categories of roads analyzed in this study (A, B, C), the mortality of badgers was 5.8 individuals per 10 km of road per whole period and there were significant differences between the road category and the number of killed badgers (χ^2^ = 12.11, *df* = 2, *p* = 0.002). Relatively higher number of killed badgers was recorded especially on secondary roads (B), than it was expected. However, the highest number of badgers that were killed was recorded on county roads (C) but it was lower than it could be expected according to high density of roads of this category (Table 1). Mean number of killed badgers was 15.3 (±5.0) per year (range: 9–20) with the lowest value in 2010 before the opening of motorway A2 and the highest in 2012 - the year of the opening of the motorway.

**Table 1:** Mortality of European badgers on roads of different categories in western Poland

Category of roads	Length of roads (km)	Proportion in road network (%)	Number of victims (N)	Proportion of victims (%)	Number of victims/10 km of road (N/10 km)
Category A	27	17.1	23	25.0	8.5
Category B	16	10.1	13	14.1	8.1
Category C	115	72.8	56	60.9	4.8
Total	158	100	92	100	5.8

**Fig. 2:**
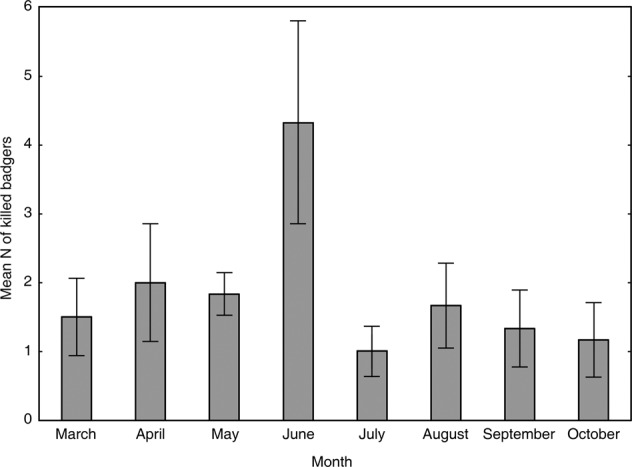
Mean number (±SE) of badger road-kills in the studied area in individual months in 2010–2015

**Fig. 3:**
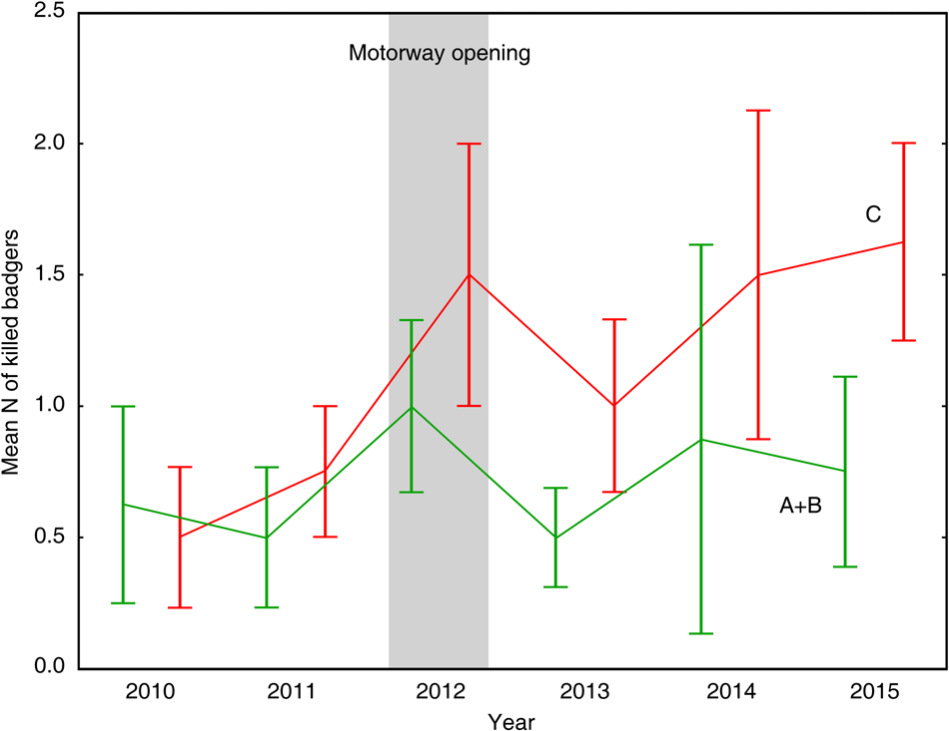
Mean number (±SE) of badger road-kills for years depending on road category (A and B data pooled) with division into two groups - before and after opening of the motorway. A, B, C - roads categories (see: Materials and methods)

There were no significant differences between the mean number of collisions with badgers between months (*F*_7, 39_ = 1.71, *p* = 0.136, Fig. 2), as well as between years (*F*_1, 39_ = 2.19, *p* = 0.147, Fig. 3). However, in 2012 the mean number of collisions reached 2.5 (±1.9) badgers per month and in following years the number of collisions was constantly higher than before 2012. When we pooled the data for seasons before (2010, 2011) and after (2012-2015) the opening of motorway, the mean number of collisions per month was significantly higher for period after 2012 on roads C (county) – hypothesis #1 partially rejected (*F*_1, 45_ = 4.29, *p* = 0.044). Year effect was not significant (*F*_1, 45_ = 0.62, *p* = 0.435). However, on roads category A + roads category B (data pooled because of low N) there were no differences in mean number of collisions per month before and after motorway opening (*F*_1, 45_ = 0.37, *p* = 0.544) – hypothesis #1 partially confirmed. Year effect was not significant (*F*_1, 45_ = 0.36, *p* = 0.549, Fig. 3). To check if other factors affected the increase in badger collisions rate in the years following motorway opening, mean number of victims was also analyzed only for period 2012-2015. However, there were no significant differences between years in period after the opening of motorway (category A + category B: *F*_1, 30_ = 0.16, *p* = 0.694 and C: *F*_1, 30_ = 0.33, *p* = 0.573).

**Fig. 4:**
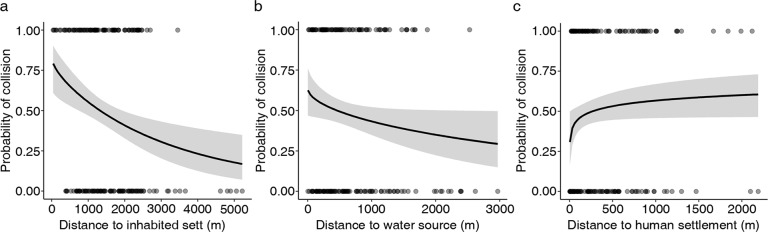
The probability of badger road-kills in relation to the distance to the nearest inhabited badger sett (**a**), water source (**b**) and human settlements (**c**) (95% confidence intervals, raw data)

Collision spots were mainly located in agricultural areas (61%), then in forests (36%) or in human settlements (3%). As a result of GLMs analysis, one of the 16 candidate models (Table S1) with environmental factors for predicting badger mortality on roads received equally high support (ΔAICc < 2). Three of the four analysed variables (distance to nearest inhabited badger sett, water source and human settlements) were found significant (Table 2). Distance to forest was not significant and did not occur in model with ΔAICc < 2 – hypothesis #2 not confirmed. The probability of badger death in road accident decreases with the increasing distance to nearest inhabited sett and water source (Fig. 4 a, b). The probability of collision increases with the growth distance to human settlements (Fig. 4 c).

**Table 2:** Model coefficients and standard errors from generalized linear model used to explain variation in the probability of collision in relation to the distance to nearest inhabited European badger sett, water source and human settlement

Variable	Estimate	*SE*	*z*	*p*
Badger sett	−0.532	0.167	−3.176	0.001
Water source	−0.474	0.175	−2.704	0.007
Human settlement	0.440	0.173	2.547	0.011

In bold *p* < 0.05

The age and sex were determined for 64 and 61 dead badgers, respectively. The sex ratio of the collision victims in total was 1:1 (male: female = 30: 31). Among females, one was pregnant and eight (26%) were nursing mothers. There were no seasonal differences (spring III-V, summer VI–VIII, autumn IX–XI) in collisions number between sexes (*χ*^2^ = 4.36, *df* = 2, *p* = 0.113) - hypothesis #3 rejected. As many as 67.2% (*N* = 43) of the victims were adult badgers and 28.1% (*N* = 18) of the victims were badgers aged 1–2 years. Only 4.7% (*N* = 3) of the victims were young individuals under one year of age.

## Discussion

The European badger is one of the most frequently road-killed mesocarnivores on European roads. Badger mortality is linked to many population and environmental factors (Aaris-Sørensen 1995; Červinka et al. 2015; Valerio et al. 2021). There is no research on the impact of a new fenced motorway on badger mortality on existing roads in the same road network. The analyses of the impact of fence construction on the existing roads indicate that they influence the increase of animal mortality on the adjacent roads with lower traffic flow (Kučas and Balčiauskas 2021). Our research results point out that opening a new, fenced motorway increases badger mortality on local roads instead of lowering it. The highest number of road-kills occurred in the year that motorway was opened. This occurred mainly in the county roads. The status of these roads has changed and now they mainly function as access roads to the motorway, especially to the motorway junction located within the research area. In the years that followed the opening of the motorway, when the new road was in constant use, we did not observe the increase in badger mortality. This absence of differences confirms that the sudden increase in 2012 was probably caused by the opening of the motorway, which affected the traffic on county roads. Despite this, we have not observed changes in the number of collisions on A and B-category roads that run in the research area, which shows that the county roads were used intensively before and after the opening of the motorway. The new road did not decrease the road traffic especially on the A-category roads, which are very important in the international transport.

Many studies present the results describing the higher badger and other carnivorous mammals mortality on local roads as compared to the roads with a higher usage status (van Langevelde et al. 2009; Dekker and Bekker 2010; Červinka et al. 2015). Our study shows that the highest number of collisions was observed on lower category roads. Higher mortality rate per kilometer of road was recorded on roads important in national and international transport.

The increased badger mortality after the opening of the motorway is mainly caused by the high death toll on the county road that connects the motorway to the main road in the region. This road is still used as a free alternative to the toll motorway, especially for the international vehicles transit traffic. The increase in mortality on county roads is also visible in the sett occupancy. After opening of the motorway in 2012, three of the six main setts in the county road vicinity were abandoned and not inhabited till the end of the research period. Badgers reinhabited one of these setts eight years later (Nowakowski K. – personal observation). Sett recolonization is a poorly studied process, but it can be assumed that the collisions affected the dispersive ability of badgers, which is related to the population density (Rogers et al. 1998). In the lowland Poland, the density of badgers is one of the lowest recorded in the literature, ranging from 1.6 individuals/10 km^2^ in the Białowieża Forest in the east (Kowalczyk et al. 2000) to 3.1 individuals/10 km^2^ in central part of the country (Goszczyński and Skoczyńska 1996) and 5.9 individuals/10 km^2^ in the northeast (Goszczyński 1999). The badger population within the area under presented study is also not very numerous and is characterized by a low number of cubs per litter (Nowakowski et al. 2020).

In contrast, the highest mortality among badgers on roads is reported in countries where badger populations are characterized by high densities and, simultaneously, the road network is highly developed. In Great Britain, where badgers are exceptionally numerous, mortality has been estimated at 50,000 individuals per year (Davies et al. 1987). The British Isles have the highest densities of badgers, 93.8 individuals/10 km^2^ on average, as compared to other regions (Kowalczyk et al. 2000) but the victims of collisions with vehicles constitute 20% of the population of adult badgers (Clarke et al. 1998). In the Netherlands, as many as 25% individuals of local populations die in collisions yearly (Jaarsma et al. 2007; van Langevelde et al. 2009; Dekker and Bekker 2010). However, the densities of these populations amount locally to 22 individuals/10 km^2^ (van Apeldoorn et al. 2006). In Sweden, where the badger population density is lower, it is estimated that 22,000–33,000 individuals die, which constitutes 11% of the population (Seiler et al. 2004). High mortality of badgers on roads has also been reported in Denmark (Aaris-Sørensen 1995). The annual death rate of badgers on the roads amounts to 20% of the total population (Dekker and Bekker 2010). Mortality of badgers on roads can be a serious factor reducing local populations.

Our results show that a significant relationship exists between the location of a collision site and the distance from the human settlements. The majority of badgers were killed on farmland areas, mainly these in close vicinity to villages. The urban habitats and farmlands are important factors increasing badger mortality on roads (Valerio et al. 2021). Badgers are known for preferring farmland-forest mosaic landscapes (Kauhala and Auttila 2010). Badgers eagerly search for food in farmlands. They are especially attracted to areas where maize is grown, as it may constitute an essential part of their diet in a mosaic landscape (Balestrieri et al. 2004).

The seasonal distribution of badger road-killing incidents in western Poland confirms data from Denmark, where the majority of badgers were also killed in June (Aaris-Sørensen 1995). In contrast to Denmark, we did not record an increase in October. In the south-west England the highest mortality occurred in spring (Forman and Alexander 1998). Only in Italy, the highest badger collision mortality rate was recorded in winter months (Valerio et al. 2021). Increased mortality during summer may be associated with the intensified badger mobility during that period. In western Poland, 80% of females were killed during the spring-summer months (March-June). Females, especially those nursing cubs, penetrate larger territories in search of food. The size of the penetrated area is also related to the food availability. In habitats with low or scattered abundance of worms, badgers cover greater distances (Kowalczyk et al. 2006). Badgers spend the least time in setts during June and July (Kowalczyk et al. 2004).

Importance of a motorway in the landscape is usually considered as an animal movement barrier and in the context of animal mortality on the motorway itself (e.g., Forman et al. 2003; Coffin 2007; McGregor et al. 2008; Ascensão et al. 2015; Ruiz-Capillas et al. 2015; Chen and Koprowski 2016; Andersson et al. 2017). The opening of a motorway usually causes a decrease in road traffic on local roads. In our study, the economic factors, such as tolls and the lack of infrastructure, determine that the lower-status road located near the motorway is still intensively used and important in transit traffic. In addition, the importance of local motorway access roads has increased. The appearance of a new, safe road in the landscape, analysed in our research, did not result in the expected decrease in animal vehicle collision mortality rate, which we observed on the example of badgers. The mortality rate of this mesopredator has even increased on the motorway access roads. In the light of our results, it seems sensible to extend the motorway animal mortality monitoring to local roads and to apply protective measures, such as fences and animal crossings, not only on the motorway, but also in its vicinity.

## Supplementary information

Table S1
