# Supplementary material for: Long Arm of Motorway—The Impact of Fenced Road on the Mortality of European Badgers
Source: Environ Manage. 2021 Nov 29;69(2):429–37. doi: 10.1007/s00267-021-01570-y (PMC8789629; doi:10.1007/s00267-021-01570-y)
Supplement: Supplementary file 1 — Table S1 [file 267_2021_1570_MOESM1_ESM.doc]

Table S1 Results of the generalized linear models used to explain variation in the probability of European badger mortality (estimate ±SE) in relation to the distance to: nearest inhabited badger sett, water source, human settlement and forest

| Model rank | Intercept | Badger sett | Water source | Human settlement | Forest | df | AICc | ΔAICc | ωAICc |
| --- | --- | --- | --- | --- | --- | --- | --- | --- | --- |
| 1 | -0.069 ± 0.1542 | -0.532 ± 0.1674 | -0.474 ± 0.1751 | 0.440 ± 0.1727 | - | 4 | 248.5 | 0.00 | 0.621 |
| 2 | -0.070 ± 0.1543 | -0.541 ± 0.1744 | -0.472 ± 0.1753 | 0.448 ± 0.1786 | 0.031 ± 0.1657 | 5 | 250.6 | 2.07 | 0.220 |
| 3 | -0.066 ± 0.1514 | -0.536 ± 0.1632 | -0.315 ± 0.1551 | - | - | 3 | 253.4 | 4.85 | 0.055 |
| 4 | -0.071 ± 0.1511 | -0.519 ± 0.1643 | - | 0.273 ± 0.1535 | - | 3 | 254.4 | 5.87 | 0.033 |
| 5 | -0.066 ± 0.1515 | -0.514 ± 0.1686 | -0.326 ± 0.1565 | - | -0.082 ± 0.1585 | 4 | 255.2 | 6.67 | 0.022 |
| 6 | -0.068 ± 0.1497 | -0.526 ± 0.1620 | - | - | - | 2 | 255.6 | 7.05 | 0.018 |
| 7 | -0.072 ± 0.1511 | -0.534 ± 0.1717 | - | 0.288 ± 0.1610 | 0.051 ± 0.1639 | 4 | 256.4 | 7.86 | 0.012 |
| 8 | -0.068 ± 0.1497 | -0.515 ± 0.1679 | - | - | -0.038 ± 0.1557 | 3 | 257.6 | 9.06 | 0.007 |
| 9 | -0.072 ± 0.1498 | - | -0.455 ± 0.1688 | 0.450 ± 0.1673 | - | 3 | 257.6 | 9.11 | 0.007 |
| 10 | -0.071 ± 0.1500 | - | -0.461 ± 0.1692 | 0.418 ± 0.1724 | -0.121 ± 0.1554 | 4 | 259.1 | 10.59 | 0.003 |
| 11 | -0.066 ± 0.1476 | - | -0.323 ± 0.1520 | - | -0.217 ± 0.1498 | 3 | 263.2 | 14.71 | 0.000 |
| 12 | -0.066 ± 0.1467 | - | -0.295 ± 0.1500 | - | - | 2 | 263.3 | 14.76 | 0.000 |
| 13 | -0.065 ± 0.1467 | - | - | 0.290 ± 0.1496 | - | 2 | 263.4 | 14.88 | 0.000 |
| 14 | -0.065 ± 0.1468 | - | - | 0.261 ± 0.1558 | -0.101 ± 0.1535 | 3 | 265.0 | 16.51 | 0.000 |
| 15 | -0.063 ± 0.1452 | - | - | - | - | 1 | 265.2 | 16.70 | 0.000 |
| 16 | -0.064 ± 0.1457 | - | - | - | -0.175 ± 0.1470 | 2 | 265.8 | 17.31 | 0.000 |
